# Parliament and the Professions

**Published:** 1921-03-12

**Authors:** 


### Parliament and the Professions.

### Proprietary Medicines Bill.

The Minister of Health was asked a question by Captain Tudor-Rees as to the protection of the public in respect of advertisements of alleged cures of cancer and other diseases. Dr. Addison said that the Proprietary Medicines Bill, introduced last Session, is being revised to meet certain objections of the commercial interests concerned, and he hoped it would be possible to reintroduce the measure during the present Session.

### Industrial Fatigue Research Board.

Captain Coote asked the Minister of Health whether he is now in a position to give a definite reply as to the continuance of the Industrial Fatigue Research Board; and whether any steps are being taken to draw the attention of the steel-making industry to the report of the Board on the subject. Mr. Mills asked a question as to the decision of the Treasury to withdraw financial support from the Board. Dr. Addison stated that the expenditure is naturally being reviewed with all other expenditure in the light of the imperative need for economy. The Board would, however, be continued, and he could promise that sufficient funds would be provided to secure its efficiency.

### Cost of Paupers and Lunatics.

The Minister of Health stated that in the last completed financial year (1919-20) the expenditure of local authorities in England and Wales on the relief of the poor and on lunatics and lunatic asylums was borne to the following extent, approximately, by rates and taxes—namely, rates, £20,900,000; taxes, £2,600,000.

### Ministry of Health Bills.

The Minister of Health said that he proposed to reintroduce the Housing Bill and the Tuberculosis Bill before Whitsuntide. He was unable at present to give information about any other measures affecting public health or local administration.

### Tuberculosis.

Lieut.-Colonel Raw asked the Minister of Health whether the exposure and hardships to which large numbers of healthy men were subjected on active service have resulted in an increase in the mortality from tuberculosis.

Dr. Addison replied that, although the conditions of active service no doubt increased the mortality from tuberculosis in weakly men and in those suffering from tuberculosis, he is advised that there is no evidence to show that there has been any increase in mortality from this disease amongst men who were healthy on entering upon naval or military service; 1919 showed a large decrease in deaths from tuberculosis in England and Wales. The figures were 46,312 for 1919, 58,073 for 1918, and 55,934 for 1917. The figures for 1920 indicate another decrease.

